# Determinants of HbA1c Variability Across Clinical Subgroups: When the Same Value Does Not Reflect the Same Biological Risk

**DOI:** 10.3390/medicina62030451

**Published:** 2026-02-27

**Authors:** Mihaela Simona Popoviciu, Timea Claudia Ghitea, Carmen Pantis, Roxana Daniela Brata

**Affiliations:** 1 Department of Preclinical Disciplines, Faculty of Medicine and Pharmacy, University of Oradea, 1 Decembrie, 410028 Oradea, Romania; mihaela.popoviciu@didactic.uoradea.ro; 2 Department of Internal Medicine II, Diabetes Mellitus, Clinical County Emergency Hospital of Oradea, 410167 Oradea, Romania; 3 Pharmacy Department, Faculty of Medicine and Pharmacy, University of Oradea, 1 University Street, 410087 Oradea, Romania; 4 Department of Medical Disciplines, Faculty of Medicine and Pharmacy, University of Oradea, 1 Decembrie, 410028 Oradea, Romania; brata.roxanadaniela@didactic.uoradea.ro

**Keywords:** HbA1c, metabolic risk, clinical subgroups, sex differences, obesity, complication burden, personalized risk assessment, diabetes mellitus

## Abstract

*Background and Objectives:* Glycated hemoglobin (HbA1c) is widely used as a marker of long-term glycemic control and metabolic risk, and represents a cornerstone in the diagnosis and management of diabetes mellitus, as endorsed by major international diabetes guidelines. However, its interpretation is typically uniform across patient populations, despite growing evidence that the biological and clinical significance of a given HbA1c value may vary depending on individual characteristics. *Materials and Methods*: In this cross-sectional observational study, 839 adult subjects from a real-world clinical cohort were analyzed to assess HbA1c variability and its association with cumulative diabetes-related complication burden (neuropathy, retinopathy, nephropathy, peripheral arterial disease), used here as a proxy for biological risk. Biological risk was assessed using the cumulative number of documented diabetes-related complications. To evaluate whether similar HbA1c values reflect comparable biological risk, comparisons were conducted within a predefined HbA1c stratum (6.0–6.9%). Linear regression and stratified analyses were used to explore context-dependent associations between HbA1c and cumulative complication burden. *Results*: HbA1c distributions showed substantial overlap across sex and obesity categories, with no marked differences in central tendency. Within the HbA1c range of 6.0–6.9%, differences in cumulative complication burden were observed across subgroups, with males and obese individuals showing a numerically higher mean number of complications despite comparable glycemic levels. Subgroup-specific regression analyses suggested heterogeneous associations between HbA1c and complication burden, indicating potential modification of HbA1c-related risk by clinical context. *Conclusions*: These findings demonstrate that the clinical interpretation of HbA1c should be contextualized. Identical HbA1c values may be associated with different complication profiles depending on sex and obesity status. Incorporating clinical context into HbA1c-based risk assessment may help inform more personalized approaches to metabolic risk stratification.

## 1. Introduction

Glycated hemoglobin (HbA1c) is widely used as a marker of long-term glycemic exposure and represents a cornerstone in the diagnosis and management of diabetes mellitus. Disease duration may further modify this relationship by influencing cumulative glycemic exposure and complication risk. Its simplicity and standardized measurement have led to broad clinical adoption, often resulting in HbA1c being interpreted as a universal indicator of metabolic risk across diverse patient populations [1,2,3,4]. Beyond diabetes management, HbA1c has also been associated with subclinical cardiovascular dysfunction even in non-diabetic populations [5].

However, accumulating evidence suggests that the biological meaning of a given HbA1c value may vary according to individual clinical characteristics. Factors such as sex, adiposity, age, and comorbid conditions can influence glucose metabolism, erythrocyte turnover, inflammatory status, and tissue susceptibility to metabolic injury. As a result, identical HbA1c values may not reflect equivalent degrees of metabolic stress or complication risk across different patient subgroups [6,7,8,9].

Sex-related differences in HbA1c interpretation have been attributed to hormonal influences, variations in red blood cell lifespan, and differences in inflammatory and oxidative stress responses. Similarly, obesity is a key modifier of metabolic risk, contributing to insulin resistance, chronic low-grade inflammation, and altered glycemic dynamics. These factors may modify the relationship between HbA1c and downstream clinical outcomes, including microvascular complications [10].

Despite these considerations, current clinical practice largely relies on uniform HbA1c thresholds for risk assessment and therapeutic decision-making. This approach may obscure clinically relevant heterogeneity, particularly in patients with intermediate or borderline glycemic values. Evaluating HbA1c variability and its clinical implications within relevant subgroups may therefore provide valuable insights into personalized risk stratification [11].

The aim of the present study was to investigate the determinants of HbA1c variability and its association with diabetes-related complication burden across clinically relevant subgroups, focusing on sex and obesity status. Using complication burden, quantified as the cumulative number of documented diabetes-related complications per patient, this study aims to highlight the importance of contextualizing HbA1c interpretation in routine clinical practice.

## 2. Materials and Methods

### 2.1. Study Design and Population

This cross-sectional observational study included adult subjects with available data on HbA1c levels, clinical characteristics, and diabetes-related complications, retrospectively collected from an institutional database between 2021 and 2024. Only participants with complete information on HbA1c, sex, obesity status, and documented complication data were included in the present analyses.

The study population represents a real-world clinical cohort with heterogeneous metabolic profiles, allowing subgroup-based evaluation of HbA1c variability and its clinical implications. The cohort was ethnically homogeneous and predominantly Caucasian/European, reflecting the regional population structure. Due to limited ethnic variability, ethnicity-based subanalyses were not feasible.

### 2.2. Clinical and Metabolic Assessment

Demographic variables included age and sex. Glycemic status was assessed using HbA1c (%) and fasting plasma glucose (mg/dL), measured according to standard laboratory procedures.

Obesity status was defined based on body mass index (BMI) and categorized as normal weight, overweight, and obesity grades I–III, in accordance with standard clinical classifications: normal weight (18.5–24.9), overweight (25–29.9), obesity I (30–34.9), obesity II (35–39.9), obesity III (≥40 kg/m^2^). For selected analyses, obesity categories were further grouped into non-obese (normal weight and overweight) and obese (grades I–III) to facilitate subgroup comparisons.

HbA1c was analyzed as a continuous variable in all primary analyses. No diagnostic categorization based on HbA1c thresholds was applied, except for predefined stratified analyses within narrow HbA1c intervals, identified from documented clinical diagnoses recorded in medical records by treating specialists. HbA1c measurements were performed in a certified clinical laboratory using standardized methods aligned with NGSP and IFCC reference systems.

### 2.3. Assessment of Diabetes-Related Complications

Diabetes-related complications were identified from documented clinical diagnoses and included peripheral neuropathy, diabetic retinopathy, diabetic nephropathy, and peripheral arterial disease. Complications were identified from documented diagnoses recorded by treating specialists in medical records. Patients were not assumed complication-free unless prior evaluation was documented. Complication status was based on documented specialist diagnoses recorded in routine clinical care. In the absence of documented evaluation, complication status was treated as not documented rather than absent.

A cumulative complication burden was calculated for each subject as the total number of documented diabetes-related complications. For subgroup comparisons, both the cumulative number of complications and the presence of at least one complication were evaluated, as appropriate.

### 2.4. Subgroup and Stratified Analyses

Subgroup analyses were performed according to sex (female vs. male) and obesity status. To assess whether similar HbA1c values were associated with different complication burdens across subgroups, analyses were restricted to a predefined HbA1c stratum of 6.0–6.9%, representing a clinically relevant intermediate glycemic range.

Within this HbA1c stratum, differences in cumulative complication burden were compared across sex and obesity categories using non-parametric statistical tests.

### 2.5. Statistical Analysis

Continuous variables are presented as mean ± standard deviation or median, as appropriate. Categorical variables are presented as number and percentage.

Group comparisons were performed using the Mann–Whitney U test for two-group comparisons (sex) and the Kruskal–Wallis test for comparisons across multiple obesity categories. Linear regression analyses were conducted within subgroups to explore context-dependent associations between HbA1c and cumulative complication burden. Regression coefficients (β) represent the estimated change in the number of complications per 1% increase in HbA1c. Interaction analyses were exploratory and not adjusted for multiple testing.

Additional sex-stratified analyses were performed to compare HbA1c levels and complication burden across obesity categories within each sex.

All analyses were considered exploratory and were interpreted accordingly. Statistical analyses were performed using IBM SPSS Statistics (version 30, IBM Corp., Armonk, NY, USA). A two-sided *p*-value < 0.05 was considered statistically significant.

## 3. Results

### 3.1. HbA1c Variability Across Sex and Obesity Subgroups

HbA1c values showed substantial variability across clinical subgroups defined by sex and obesity status. Within HbA1c 6.0–6.9%, males showed a higher mean complication count than females (0.81 vs. 0.69), although this difference did not reach statistical significance (*p* = 0.133). Median HbA1c values were comparable between sexes (6.8% (IQR 6.2–7.4) in females vs. 7.0% (IQR 6.4–7.8) in males), indicating overlapping glycemic distributions (Table 1).

**Figure 1:**
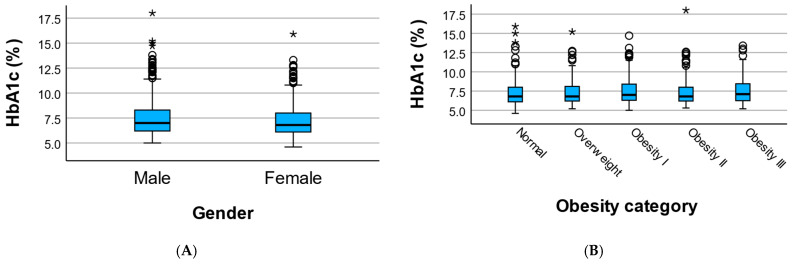
HbA1c distribution by sex (**A**) and across obesity categories (**B**). Boxplot illustrating the distribution of HbA1c values by sex and the distributions across BMI-defined obesity categories. The central line represents the median, the box indicates the interquartile range, and individual points represent outliers. Despite overlapping medians, greater dispersion and higher extreme values are observed in obese subgroups. Circles (o) indicate mild outliers (>1.5×IQR), and asterisks (*) indicate extreme outliers (>3×IQR).

Across obesity categories, HbA1c values demonstrated heterogeneous distributions. Mean HbA1c tended to be higher in individuals with obesity, particularly in those classified as obesity grade I and III; however, overall differences across BMI-defined categories were not statistically significant (Kruskal–Wallis *p* = 0.477). Despite similar median values across groups, wider dispersion and higher extreme values were observed in obese subgroups (Figure 1).

These findings indicate that similar HbA1c values may occur across different clinical contexts, with substantial overlap between sex- and obesity-defined subgroups, suggesting that HbA1c interpretation should be contextualized rather than viewed as a uniform marker of metabolic risk (Table 2).

### 3.2. Differential Complication Burden at Similar HbA1c Levels

To evaluate whether similar HbA1c values reflect comparable biological risk across clinical subgroups, analyses were performed within a predefined HbA1c stratum of 6.0–6.9%, corresponding to a frequently encountered intermediate glycemic range.

Within this HbA1c interval, the cumulative burden of diabetes-related complications differed modestly across sex subgroups. Male participants exhibited a slightly higher mean number of complications compared to females (0.81 vs. 0.69), although this difference did not reach statistical significance (*p* = 0.221). Median values were identical in both groups (median = 1), indicating overlapping complication distributions despite similar glycemic exposure.

When stratified by obesity status, individuals with obesity—particularly those classified as obesity grade I and II—tended to present a higher mean complication burden compared to normal-weight subjects (0.83–0.76 vs. 0.64). However, differences across obesity categories were not statistically significant (Kruskal–Wallis *p* = 0.116). Notably, variability within obesity subgroups was substantial, with overlapping medians and wide dispersion of complication counts.

Overall, these findings indicate that patients with comparable HbA1c values may exhibit different degrees of complication burden depending on sex and adiposity, supporting the concept that HbA1c interpretation should be contextualized within the clinical profile rather than applied uniformly (Table 3).

**Figure 2:**
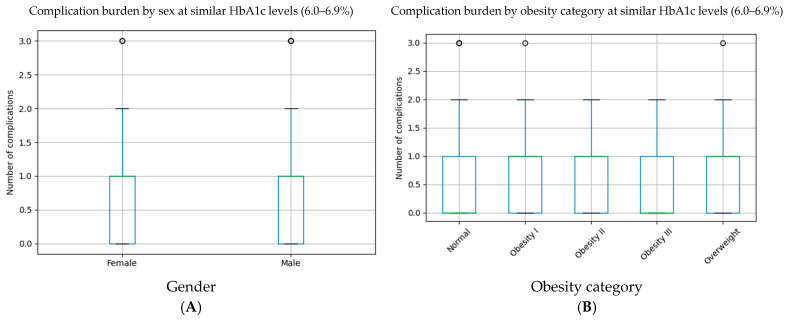
Differential complication burden at similar HbA1c levels (6.0–6.9%). (**A**) Complication burden by sex. Boxplot illustrating the distribution of the cumulative number of diabetes-related complications in female and male subjects within the HbA1c range of 6.0–6.9%. Despite comparable HbA1c values, variability in complication burden is observed between sexes. (**B**) Complication burden by obesity category. Boxplot illustrating the distribution of the cumulative number of diabetes-related complications across BMI-defined obesity categories among subjects with HbA1c values between 6.0% and 6.9%. Overlapping medians and wide dispersion highlight heterogeneous complication burden at similar glycemic levels.

The complication burden at similar HbA1c levels (6.0–6.9%) is presented in Figure 2.

### 3.3. Distribution of Outcomes by Sex Within Obesity Groups

Sex-stratified analyses were conducted to evaluate HbA1c levels and complication burden across BMI-defined obesity categories.

Among females, mean HbA1c values were comparable across obesity groups (7.42–7.99%). The cumulative complication burden showed modest variation but no consistent gradient. Kruskal–Wallis testing indicated no significant differences across obesity categories for either HbA1c (*p* = 0.605) or complication burden (*p* = 0.552).

Among males, mean HbA1c ranged from 7.15% to 7.55% across obesity categories. Complication burden tended to be higher in obesity grade III; however, differences across categories were not statistically significant. Kruskal–Wallis testing again showed no significant differences for HbA1c (*p* = 0.122) or complication burden (*p* = 0.122).

Overall, these findings suggest broadly similar HbA1c and complication patterns across obesity categories within each sex, without statistically significant within-sex differences (Table 4).

### 3.4. Interaction Effects Between HbA1c and Clinical Context

To formally assess whether the association between HbA1c and complication burden is modified by clinical context, interaction analyses were performed between HbA1c and sex, as well as between HbA1c and obesity status.

Interaction models suggested that the relationship between HbA1c and cumulative complication burden differed modestly across sex subgroups. While increasing HbA1c values were associated with higher complication burden in both sexes, the slope of this association appeared slightly steeper in male subjects compared to females, indicating a potentially greater sensitivity to glycemic exposure in males at comparable HbA1c levels. However, the interaction term did not reach statistical significance, reflecting overlapping risk patterns between sexes.

Similarly, analyses exploring the interaction between HbA1c and obesity status indicated heterogeneous risk trajectories across BMI-defined categories. In obese individuals, higher HbA1c values tended to be associated with a more pronounced increase in complication burden compared to normal-weight subjects, suggesting that adiposity may amplify the biological impact of chronic hyperglycemia. Nevertheless, interaction terms did not reach formal statistical significance, likely due to limited power within individual subgroups.

Although β coefficients were numerically small, they represent incremental changes per 1% HbA1c increase and should be interpreted in the context of cumulative long-term glycemic exposure.

Overall, although interaction effects were modest, these findings support the concept that HbA1c does not convey identical biological meaning across different clinical contexts. Sex and obesity appear to influence the expression of complication risk at similar HbA1c levels, reinforcing the need for contextualized interpretation of glycemic markers in clinical practice (Table 5).

## 4. Discussion

In this study, we explored the clinical determinants of HbA1c variability and its association with complication burden across relevant patient subgroups, focusing on sex and obesity as key modifiers. Our findings indicate that identical HbA1c values do not necessarily reflect equivalent biological or clinical risk, emphasizing the importance of contextualized interpretation of glycemic markers.

### 4.1. HbA1c Variability Across Clinical Subgroups

We observed substantial overlap in HbA1c distributions across sex and obesity categories, with no marked differences in central tendency. However, greater dispersion and higher extreme values were observed in certain subgroups, particularly among obese individuals. These findings suggest that HbA1c variability is influenced by underlying clinical context rather than being a uniform marker across populations [12,13,14,15,16].

Sex-related differences in HbA1c interpretation may reflect biological factors such as erythrocyte turnover, hormonal influences, and differences in inflammatory or oxidative stress profiles. Similarly, obesity is associated with insulin resistance, chronic low-grade inflammation, and altered glucose metabolism, which may modify the relationship between HbA1c and downstream metabolic consequences [17,18,19,20].

### 4.2. Variation in Complication Burden at Equivalent Glycemic Control

A central finding of this study is that patients with comparable HbA1c values can exhibit different degrees of complication burden depending on sex and obesity status. Within a narrow HbA1c range (6.0–6.9%), males and individuals with obesity tended to show a higher cumulative number of complications compared to their counterparts, despite similar glycemic exposure [21,22,23,24].

These observations reinforce the concept that HbA1c alone may be insufficient to fully characterize metabolic risk. Instead, HbA1c should be interpreted alongside clinical modifiers that influence tissue susceptibility and cumulative metabolic damage [25].

### 4.3. Context-Dependent Associations and Interaction Effects

Exploratory interaction analyses further suggested that the association between HbA1c and complication burden may differ across clinical contexts. Subgroup-specific regression analyses indicated varying slopes linking HbA1c to complication burden by sex and obesity status, supporting the hypothesis that the biological meaning of HbA1c is not constant across patient populations [26,27,28,29].

Although interaction terms did not reach formal statistical significance, the consistency of directional trends across analyses suggests potential effect modification that warrants further investigation in larger, prospectively designed cohorts.

### 4.4. Clinical Implications

From a clinical perspective, these findings highlight the limitations of a “one-size-fits-all” approach to HbA1c interpretation. Patients with similar HbA1c values may require different levels of surveillance and intervention depending on sex, adiposity, and overall clinical profile. Integrating subgroup-specific context into glycemic assessment may improve risk stratification and support more personalized clinical decision-making [30,31,32,33].

### 4.5. Strengths and Limitations

The strengths of this study include the use of a real-world clinical cohort, the focus on clinically relevant subgroups, and the integration of descriptive, stratified, and interaction-based analytical approaches. However, several limitations should be acknowledged. The cross-sectional design limits causal inference, and subgroup analyses may have been underpowered to detect statistically significant interaction effects. Additionally, unmeasured confounders, such as disease duration, treatment intensity, and lifestyle factors, may have influenced observed associations.

In summary, our findings suggest that the interpretation of HbA1c should be contextualized within the clinical profile of the patient. Sex and obesity may modify the relationship between HbA1c and complication burden, supporting the concept that the same HbA1c value does not equate to the same biological risk across individuals. Not all participants underwent systematic screening for each complication. Future studies should further investigate subgroup-specific HbA1c thresholds and their implications for personalized metabolic risk assessment.

## 5. Conclusions

This study demonstrates that HbA1c variability and its association with complication burden are influenced by clinical context. Sex and obesity appear to influence the relationship between HbA1c and metabolic risk, indicating that identical HbA1c values may reflect different biological and clinical profiles across patient subgroups.

Our findings support the concept that HbA1c should not be interpreted in isolation. Incorporating subgroup-specific clinical characteristics may improve the assessment of metabolic risk and help identify individuals who require closer monitoring or earlier intervention despite similar glycemic values.

Overall, these results underscore the potential need for a more personalized approach to glycemic assessment, in which HbA1c is interpreted within the broader clinical context rather than as a uniform risk marker.

## Figures and Tables

**Table 1:** Baseline characteristics of the study cohort (*n* = 839).

Variable	Value
Age (years)	
Mean ± SD	63.11 ± 10.23
Median (IQR)	64 (56–70)
Min–Max	27–90
Sex, *n* (%)	
Male	432 (51.5%)
Female	407 (48.5%)
HbA1c (%)	
Mean ± SD	7.43 ± 1.79
Median (IQR)	6.9 (6.2–8.1)
Min–Max	4.6–18.3

Data are presented as mean ± standard deviation or median (interquartile range), as appropriate. Categorical variables are presented as number (percentage). HbA1c values were obtained from routine clinical measurements performed in a certified laboratory aligned with NGSP/IFCC standards. HbA1c, glycated hemoglobin; SD, standard deviation; IQR, interquartile range; NGSP, National Glycohemoglobin Standardization Program; IFCC, International Federation of Clinical Chemistry and Laboratory Medicine.

**Table 2:** Distribution of HbA1c across sex and obesity categories.

Parameter	*n*	Mean ± SD (%)	Median (IQR)	Min–Max
Sex				
Female	407	7.97 ± 6.41	7.0 (6.2–8.3)	5.0–18.3
Male	432	7.32 ± 1.65	6.8 (6.1–8.0)	4.6–15.9
*p*-value 0.133				
Obesity category				
Normal weight	192	7.33 ± 1.83	6.8 (6.1–8.0)	4.6–15.9
Overweight	198	7.35 ± 1.70	6.8 (6.2–8.1)	5.2–15.2
Obesity grade I	270	8.12 ± 7.70	7.0 (6.3–8.5)	5.0–18.3
Obesity grade II	124	7.42 ± 1.88	6.8 (6.2–8.0)	5.3–18.0
Obesity grade III	55	7.82 ± 2.15	7.1 (6.3–8.5)	5.2–13.4
*p*-value 0.477				

HbA1c values are presented as mean ± standard deviation and median. Group comparisons were performed using the Mann–Whitney U test (sex) and Kruskal–Wallis test (obesity categories).

**Table 3:** Cumulative complication burden within the HbA1c 6.0–6.9% stratum.

Parameters	*n*	Mean Number of Complications	Median
Sex			
Female	158	0.69	1
Male	136	0.81	1
*p*-value 0.221			
Obesity category			
Normal weight	64	0.64	0
Overweight	75	0.79	1
Obesity grade I	94	0.83	1
Obesity grade II	45	0.76	1
Obesity grade III	16	0.44	0
*p*-value 0.116			

Analyses were restricted to participants with HbA1c values between 6.0% and 6.9%. Data are presented as mean and median number of complications. Group comparisons were performed using the Mann–Whitney U test (sex) and the Kruskal–Wallis test (obesity categories).

**Table 4:** Sex-stratified analysis across obesity categories.

Obesity Category	*n*	Mean HbA1c ± SD	Mean Complications	KW *p*-Value (HbA1c)	KW *p*-Value (Complications)
Female					
Normal weight	95	7.50 ± 1.89	0.83	0.605	0.552
Overweight	97	7.56 ± 1.75	0.88		
Obesity I	123	7.53 ± 1.86	0.81		
Obesity II	58	7.42 ± 2.19	0.74		
Obesity III	34	7.99 ± 2.24	0.65		
Male					
Normal weight	97	7.15 ± 1.76	0.81	0.122	0.122
Overweight	101	7.15 ± 1.63	0.86		
Obesity I	147	7.46 ± 1.55	0.68		
Obesity II	66	7.42 ± 1.58	0.79		
Obesity III	21	7.55 ± 2.03	1.19		

Data are presented as mean ± standard deviation (SD) for HbA1c and as mean number of complications per patient. Obesity categories were defined according to body mass index (BMI) as follows: normal weight (18.5–24.9 kg/m^2^), overweight (25.0–29.9 kg/m^2^), obesity grade I (30.0–34.9 kg/m^2^), obesity grade II (35.0–39.9 kg/m^2^), and obesity grade III (≥40 kg/m^2^). Kruskal–Wallis (KW) tests were performed within each sex to compare HbA1c levels and cumulative complication burden across obesity categories. Reported *p*-values correspond to overall between-category comparisons within females and within males, respectively. HbA1c, glycated hemoglobin; SD, standard deviation; BMI, body mass index.

**Table 5:** Context-dependent associations between HbA1c and cumulative complication burden.

Subgroup	*n*	β (Slope) *	r	*p*-Value
Sex				
Female	432	0.046	0.095	0.048
Male	407	0.013	0.106	0.032
Obesity status				
Normal/Overweight	390	0.050	0.110	0.030
Obese (I–III)	449	0.014	0.105	0.026

* β represents the estimated increase in the number of complications per 1% increase in HbA1c. Subgroup-specific linear regression analyses were performed to explore potential interaction effects between HbA1c and clinical context. Results are presented descriptively and should be interpreted as exploratory.

## Data Availability

The data presented in this study are available on request from the corresponding author. The data are not publicly available due to privacy or ethical restrictions.

## Abbreviations

The following abbreviations are used in this manuscript:

Abbreviation	Definition
APC	Article Processing Charge
BMI	Body Mass Index
CI	Confidence Interval
HbA1c	Glycated Hemoglobin
IFCC	International Federation of Clinical Chemistry and Laboratory Medicine
IQR	Interquartile Range
KW	Kruskal–Wallis
NGSP	National Glycohemoglobin Standardization Program
OR	Odds Ratio
SD	Standard Deviation
